# Prevalence and treatment of fragility fractures in Spanish primary care: PREFRAOS study

**DOI:** 10.1007/s11657-022-01124-7

**Published:** 2022-07-15

**Authors:** Daniel Martínez-Laguna, Cristina Carbonell, José-Carlos Bastida, Milagros González, Rafael M. Micó-Pérez, Francisco Vargas, Mónica Balcells-Oliver, Laura Canals, on behalf of the PREFRAOS Group

**Affiliations:** 1 Health Center Sant Martí de Provençals, C/Fluvià 211, 08020 Barcelona, Spain; 2 GREMPAL Research Group, IDIAP Jordi Gol, Barcelona, Spain; 3 Health Center Vía Roma, Barcelona, Spain; 4 Health Center of Marín, Pontevedra, Spain; 5 Health Center of Montesa, Madrid, Spain; 6 Health Center of Fontanars dels Alforins, EAP Ontinyent, Valencia, Spain; 7 Health Center Dr. Guigou, Santa Cruz de Tenerife, Spain; 8 Medical Department, Amgen, Barcelona, Spain; 9 Medical Department, Amgen, Switzerland

**Keywords:** Fragility fracture, Osteoporosis, Primary care, Prevalence, Diagnostic, Treatment

## Abstract

**Summary:**

In Spanish primary care (PC), the prevalence of fragility fractures (FF) in subjects ≥ 70 years old is high, especially in women. One-third of subjects with an FF lacked osteoporosis (OP) diagnosis and >50% were not currently receiving OP medication. An improvement of the FF management in this population is needed.

**Purpose:**

In Spanish PC, the prevalence of FF is high, especially in women. One-third of subjects with a FF lacked an OP diagnosis and more than half were not currently receiving OP medication. Several studies reported underdiagnosis/undertreatment of OP in PC among elderly subjects with FF. To date, no such data exist for Spain. The purpose is to estimate the prevalence of FF in the elderly population (≥ 70 years old) and to describe the characteristics, risk factors, comorbidities, and OP diagnosis and treatment rates of subjects with FF in Spanish PC centers.

**Methods:**

This is an observational, retrospective study in Spain consisting of two phases. Phase A included all subjects ≥ 70 years old listed in the center’s medical records from November 2018 to March 2020. Phase B included subjects with FF and prior consultation at the center for any reason. Subjects were excluded only if they had previously participated in another study. Primary outcomes were prevalence of FF (phase A) and characteristics of subjects with at least one FF (phase B).

**Results:**

The overall prevalence of FF was 17.7% among subjects visiting medical centers for any reason (24.1% women vs. 8.0% men) (30 PC centers from 14 Spanish regions). Vertebral (5.1%) was the most prevalent fracture. Of 665 subjects in phase B, most (87%) were women and ≥ 80 years old (57%), suffered mainly major OP fracture (68%), and had multiple comorbidities (≥ 2, 89.2%). While two-thirds had OP diagnosis and 61.1% received OP medication anytime in the past, 56.8% were not currently receiving OP medication. Diagnosis and treatment rates were lower among men (43% and 38% vs. 70% and 65%, respectively).

**Conclusion:**

Prevalence of FF was high, especially in women. One-third of subjects lacked OP diagnosis and ≥ 50% were not receiving OP treatment; diagnosis and treatment gaps were larger among men. This reinforces the need to improve the management of FF in the elderly population. However, as PC centers participating in this study had high OP experience that have the potential to do better in terms of diagnosis and treatment, caution in the generalization of these data should be taken.

**Supplementary Information:**

The online version contains supplementary material available at 10.1007/s11657-022-01124-7.

## Introduction

The global incidence of osteoporosis (OP) was estimated to be over 200 million in 2020, increasing to 1.55 billion by 2050 [1]. Hence, OP has become a major problem with an enormous health and economic impact [2]. Furthermore, the probability of a fragility fracture increases with age, reaching 40% or more in subjects from Western Europe aged 50 years old [3]. While OP affects both genders, women are at higher risk of fragility fracture than men. In Spain, the probability of a fragility fracture is close to 10% in women with a fracture prior at 65 years old and increases considerably with age from 70 years old onwards [4].

Most of the elderly population with fragility fractures are undiagnosed with OP [5–7]. Furthermore, the majority of fragility fractures are not considered to be caused by bone fragility, leading to an underestimation of the true prevalence of fragility fractures [8, 9]. Additionally, the presence of bone fragility is often associated with the absence of symptoms and only one-third of vertebral fractures are symptomatic [10].

Recent studies in Spain indicate that diagnosis and treatment of OP remain suboptimal in subjects with a fragility fracture. In a retrospective study of 161 subjects with hip fracture, 14% of subjects (median age 87 years) were diagnosed with OP and only 7% received OP treatment [11]. Similarly, in a prospective, multicentric study of 487 subjects (mean age 83.1 years), 22% of subjects had a prior non-hip low-trauma fracture, 16% received osteoporotic treatment, and only 3% had densitometry performed [12].

Hence, improved identification and assessment by primary care (PC) is crucial for the management of OP [13, 14]. However, there is still a lack of knowledge when deciding whether to treat or not [15].

There are limited data regarding prevalence of fragility fractures among the elderly population (≥ 70 years) in the Spanish PC setting [16]. In a retrospective study of women indicated for densitometry, incidence of fragility fractures in women without prior fragility fractures ranged from 11/1000 person-years in those <55 years old to 55/1000 person-years in those ≥ 75 years old. The overall prevalence of fragility fractures after a median follow-up of 3.5 years was 7% [17]. Furthermore, there are few data regarding prevention, diagnosis, and treatment of OP in subjects with documented fragility fractures. According to the current National Osteoporosis Foundation [18] and SEIOMM [19] guidelines, all such subjects should be treated with at least one OP therapy. However, few data regarding the degree of compliance with these recommendations in clinical practice exist [14]. The sociodemographic and clinical profile of this population is also unknown.

Our retrospective, observational study aimed to estimate the prevalence of fragility fractures in the elderly population (≥ 70 years old) seen in Spanish PC centers, and to describe the characteristics, risk factors, comorbidities, and OP diagnosis and treatment rates of subjects with fragility fracture.

## Methods

### Study design

PREFRAOS (the PREvalence of FRAgility fractures and OSteoporosis treatment study) was an observational, retrospective, single-country chart review conducted in 30 Spanish PC centers distributed around Spain in 14 regions (Andalusia, Asturias, Canary Islands, Cantabria, Castilla la Mancha, Castile and Leon, Catalonia, Valencian Community, Estremadura, Galicia, Madrid, Murcia, Navarre, and Basque Country). Centers were chosen from those presenting a physician with a special interest in OP (such as physicians related to bone societies; or OP working groups in PC Societies or attending bone trainings). A previous feasibility study questionnaire was conducted that included information about how much easier the access to the medical records was, how many patients ≥ 70 years old were cared for by the physician, how many had fractures coded, and/or previous experience in observational studies.

The most relevant aspects included in the General Spanish Health Law of 1986 are specified in (1) universal health care. It covers 100% of the population, regardless of their economic situation and their social security affiliation; (2) public financing of assistance through taxes; (3) universal access for all citizens; (4) free benefits at the time of receiving them; and (5) primary health care is the basis of the overall health care system.

In Spain, primary health care services are organized in PC districts that make up territorial demarcations called basic health zones. Primary care centers are located in each basic health area, where PC health care is provided to citizens. Thus, they provide assistance to the entire assigned population. Primary health care in Spain is the gateway to the hospital system. In case of doubt, there is always the possibility to refer the subject to any other specialist (either in the same health center, if available, or to the referral hospital).

The study comprised two phases (A and B). The objective of phase A was to estimate the prevalence of fragility fractures in the population (women and men) ≥ 70 years old seen in Spanish PC. The objective of phase B was to describe the sociodemographic characteristics, comorbidities, risk factors for fracture, and OP diagnosis and treatment among subjects in phase A who had at least one fragility fracture, and to describe the locations and circumstances of these fractures (related to a fall). The only exclusion criterion was having previously participated in another study. This study was conducted according to the standards of the Declaration of Helsinki principles and its later amendments, and with Good Clinical Practice guidelines. All subjects provided informed consent written or oral before enrolment.

### Study population

Phase A included all subjects ≥ 70 years old listed in the investigator’s medical records between November 2018 and March 2020. Phase B included subjects from phase A with a recorded fragility fracture and prior consultation at the center for any reason. Fragility fractures were identified using International Classification of Diseases (ICD) codes (ICD-9 and ICD-10) and/or open fields, according to the characteristics of each center’s database.

At the study level, all centers were included in the study between November 2018 and January 2020 (data collection period). All data were retrospectively collected from subjects’ medical charts. There was no limit for the retrospective index period (i.e., all lifespan data will be reviewed to be able to capture all fragility fractures).

### Data collection

For phase A, each center reported the total number of subjects (overall, men, and women) and the total number of subjects ≥ 70 years old (overall, men, and women) listed in the center’s electronic medical records at the time of data collection. For subjects ≥ 70 years old, the center then retrospectively collected the total number with at least one fragility fracture—hip fracture, vertebral fracture, wrist/forearm fracture, and humerus fracture—documented. This allowed calculation of the prevalence of fragility fractures in the PC setting.

For phase B, the following variables were obtained retrospectively from subjects’ medical records: (1) sociodemographic variables: age, gender, education, marital status; (2) risk factors for fracture at the time of data collection (or last information available in the medical records): body mass index (BMI), history of falls, history of parental hip fracture, current smoking, alcohol intake ≥ 3 units/day, rheumatoid arthritis, secondary OP (all diseases considered in the FRAX^©^ tool), and associated medications (oral glucocorticoids, aromatase inhibitors, GnRH analogs, anticonvulsants, proton-pump inhibitors, antihypertensive drugs, and statins); (3) other comorbidities; (4) OP diagnosis; (5) bone mineral density (BMD): if *T*-score available and last *T*-score, location; and (6) OP treatments (previous and current). Height measurements were not collected in the subject’s follow-up, so the estimation of putative asymptomatic vertebral fracture occurrence could not be performed. Moreover, information about morphometric vertebral fractures through X-rays at fracture occurrence was also not collected.

All data were retrospectively collected from subjects’ medical charts. There was no limit for the retrospective index period (i.e., all lifespan data will be reviewed to be able to capture all fragility fractures). Subjects lost during the follow-up were not collected as there were no limits for the retrospective index period.

### Statistical analysis

All analyses were descriptive. For categorical variables, the frequency and percentage with 95% confidence interval (CI) are presented. For continuous variables, summary statistics included the number of subjects, mean, median, standard deviation (SD) or standard error (SE), 25th percentile (Q1), 75th percentile (Q3), minimum, and maximum. All data analyses were performed using SAS version 9.4 (SAS Institute, Cary, NC, USA).

In phase A, prevalence of fragility fractures was calculated using the mean number of subjects registered at each PC center in Spain as the denominator. Specifically, we estimated 476 subjects ≥ 70 years old per center (total of 19,040 subjects). This sample size offered a maximum margin of error (minimum precision) of 0.7 with maximum indetermination (*p*=*q*=50) for a 95% CI.

In phase B, a sample size of 720 subjects were chosen to offer a maximum margin of error (minimum precision) of 7.3% for categorical variables summarized as percentages. Assuming missing/incomplete data in 10% of subjects, the total sample was estimated to be 800 subjects. Finally, a total of 665 subjects were included in phase B. This sample size did not affect the intended power of the study.

## Results

### Prevalence of fragility fractures

**Fig. 1:**
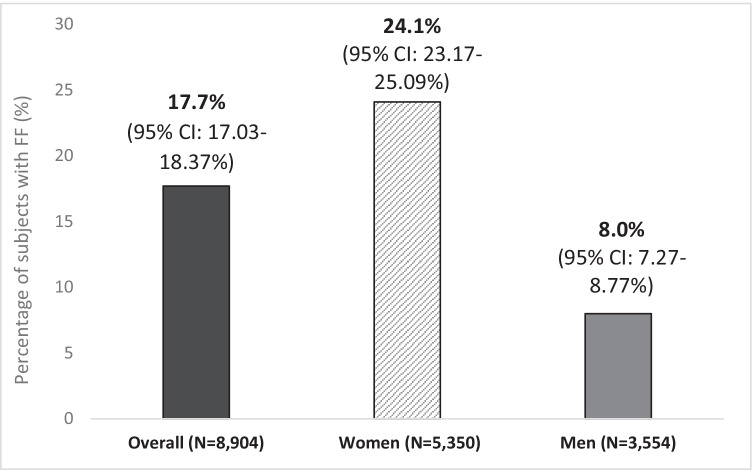
Prevalence of fragility fractures (any type) in subjects aged ≥ 70 years (phase A)

**Fig. 2:**
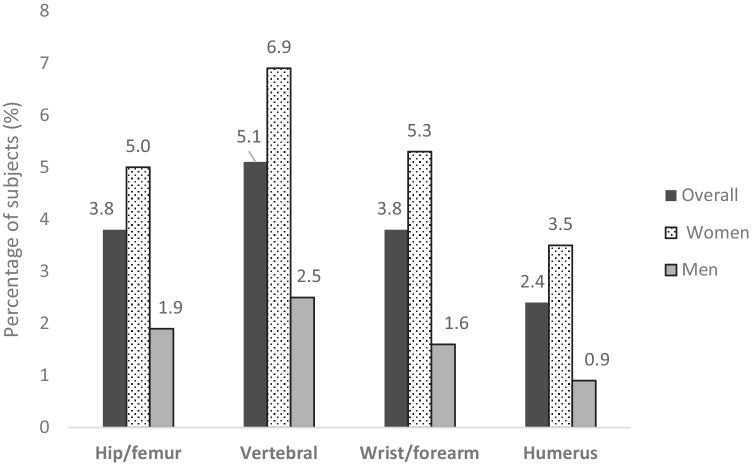
Prevalence of fragility fractures at specific sites in subjects aged ≥ 70 years (phase A)

A total of 30 PC centers from 14 Spanish regions participated in the study. From 44,062 medical records dated between November 2018 and March 2020, 8904 (20.2%) subjects were ≥ 70 years old and eligible for phase A. Of these, 17.7% had a recorded fragility fracture, with prevalence approximately 3-fold higher in women compared with that in men (24.1% vs. 8.0%, respectively) (Fig. 1). Overall, vertebral fracture was the most common OP fracture reported (5.1%) (all vertebral fractures reported in the subject file were collected) (Fig. 2).

### Demographic characteristics

Baseline characteristics of subjects included in phase B are described in Table 1. Of the 665 subjects eligible for phase B, most were women (87%) and the majority were aged ≥ 80 years old (56.7%). Mean (SD) BMI was 27.9 (4.8) kg/m^2^ (normal weight range, 25.0–29.9 kg/m^2^) [20]. The majority of subjects (70.4%) reported ≥ 4 risk factors for fracture, with history of falls (57.3%) and age ≥ 80 years (56.7%) the most common. Of the most recently reported fragility fractures, major OP fracture (hip, spine, wrist/forearm, or proximal humerus; 68.1%) was the most common.

**Table 1:** Characteristics of subjects aged ≥ 70 years with a fragility fracture (phase B)

	Overall (*n*=665)	Women (*n*=576)	Men (*n*=98)
Age (years), mean (SD)	81.9 (6.6)	81.6 (6.5)	83.2 (6.9)
Age range (years), *n* (%)			
70–74	116 (17.4)	102 (18.0)	14 (14.3)
75–79	172 (25.9)	149 (26.3)	23 (23.5)
≥ 80	377 (56.7)	316 (55.7)	61 (62.2)
BMI (kg/m^2^), mean (SD)	27.9 (4.8)	28.0 (5.0)	27.8 (4.0)
BMI range, *n* (%)			
Underweight	6 (1.1)	0 (0.0)	6 (0.9)
Normal weight	164 (28.9)	26 (26.5)	190 (28.6)
Pre-obesity	232 (40.9)	42 (42.9)	274 (41.2)
Obesity class I	114 (20.1)	26 (26.5)	140 (21.1)
Obesity class II	45 (7.9)	4 (4.1)	49 (7.4)
Obesity class III	6 (1.1)	0 (0.0)	6 (0.9)
Education, *n* (%)			
Primary school	419 (63.0)	372 (65.6)	47 (48.0)
High school/professional	94 (14.1)	76 (13.4)	18 (18.4)
University	50 (7.5)	31 (5.5)	19 (19.4)
None	102 (15.3)	88 (15.5)	14 (14.3)
Marital status, *n* (%)			
Single	64 (9.6)	60 (10.6)	4 (4.1)
Married	259 (38.9)	194 (34.2)	65 (66.3)
Widowed	324 (48.7)	299 (52.7)	25 (25.5)
Divorced	18 (2.7)	14 (2.5)	4 (4.1)
BMI <19 kg/m^2^	8 (1.2)	8 (1.4)	0 (0.0)
History of falling	381 (57.3)	327 (57.7)	54 (55.1)
Age ≥ 80 years	377 (56.7)	316 (55.7)	61 (62.2)
Father/mother with hip fracture	40 (6.0)	37 (6.5)	3 (3.1)
Secondary OP	37 (5.6)	26 (4.6)	11 (11.2)
Rheumatoid arthritis	18 (2.7)	11 (1.9)	7 (7.1)
Use oral glucocorticoids, *n* (%)	24 (3.6)	17 (3.0)	7 (7.1)
Smoker, *n* (%)	19 (2.9)	12 (2.1)	7 (7.1)
Alcohol (≥ 3 units/day), *n* (%)	21 (3.2)	11 (1.9)	10 (10.2)

*BMI*, body mass index; *OP*, osteoporosis; *SD*, standard deviation

### Other characteristics

Most subjects (90%) had two or more comorbidities, the most common (occurring in ≥ 20% of subjects) being arthrosis (73.7%), hypertension (70.5%), anxiety (33.8%), sleep disorders (31.1%), depression (29.9%), and diabetes (20.8%). Furthermore, 14% of subjects had chronic kidney disease (CKD), of which 44.1% had stage 3A and 31.2% stage 3B (Table 2).

**Table 2:** Comorbidities of subjects aged ≥ 70 years with a fragility fracture (phase B)

	Overall (*n*=665)	Women (*n*=576)	Men (*n*=98)
Comorbidities, *n* (%)			
0	12 (1.8)	9 (1.6)	3 (3.1)
1	60 (9.0)	52 (9.2)	8 (8.2)
2–4	383 (57.6)	338 (59.6)	45 (45.9)
≥ 5	210 (31.6)	168 (29.6)	42 (42.9)
Major comorbidities (yes), *n* (%)			
Arthrosis	490 (73.7)	420 (74.1)	70 (71.4)
Hypertension	469 (70.5)	401 (70.7)	68 (69.4)
Anxiety	225 (33.8)	201 (35.4)	74 (75.5)
Sleep disorders	207 (31.1)	180 (31.7)	27 (27.6)
Depression	199 (29.9)	173 (30.5)	26 (26.5)
Type 2 diabetes	138 (20.8)	112 (19.8)	26 (26.5)
Chronic obstructive pulmonary disease	66 (9.9)	43 (7.6)	23 (23.5)
Rheumatoid arthritis	18 (2.7)	11 (1.9)	7 (7.1)
Chronic kidney disease	93 (14)	74 (13.1)	19 (19.4)
Chronic kidney disease staging, *n* (%)			
Stage 1	0 (0.0)	0 (0.0)	0 (0.0)
Stage 2	14 (15.1)	10 (13.5)	4 (21.1)
Stage 3A	41 (44.1)	32 (43.2)	9 (47.4)
Stage 3B	29 (31.2)	24 (32.4)	5 (26.3)
Stage 4	9 (9.7)	8 (10.8)	1 (5.3)

### OP diagnosis and treatment

Overall, two-thirds (65.7%) of subjects included in phase B had an OP diagnosis; compared with women, men had lower diagnosis rates (69.7% vs. 42.9%). While 61.1% of all subjects had received OP medication at any time, with the majority (62.1%; Table S1) having received only one OP treatment, more than half of all subjects (56.8%) were not receiving OP treatment at inclusion into phase B (Table 3). This treatment gap was higher among men than that in women (71.4% vs. 54.3%; Table 3). Compared with subjects with an OP diagnosis, the treatment gap was twofold higher in subjects without an OP diagnosis (86.8% vs. 41.2%; Table S2). *T*-score was available in 41.5% of all subjects, and twice as likely to be available in women than that in men (44.8% [254/567] vs. 22.4% [22/98], respectively).

**Table 3:** OP diagnosis and treatment among subjects aged ≥ 70 years with fragility fractures (phase B)

Percentage of subjects with FF (%)	Women (*n*=576)	Men (*n*=98)	Overall (*n*=665)
OP diagnosis	69.7	42.9	65.7
OT treatment at any time	65.1	37.8	61.1
OP treatment at study entry	45.7	28.6	43.2
OP treatment at study entry in subjects with OP diagnosis	59.5	52.4	58.8

*FF*, fragility fracture

### Fracture characteristics

Among the 665 subjects included in part B, 928 fractures (800 fractures in women and 128 in men) were reported. Overall, the most common fracture type was vertebral (32.9%), with similar incidence among men and women (32% and 33%, respectively), followed by hip/femur (18.8%) and humerus (12.3%). The most common fracture type among women was vertebral (33%) compared with hip/femur among men (36.7%) (Table S3). In most subjects, their fracture was reported to be related to a “fall” (women, 603/800 [75.4%]; men, 98/128 [76.6%)]); mean (SD) age at fracture was comparable for men and women (79.0 [9.6] vs. 76.1 [8.4] years). Mean age of subjects when fragility fractures occurred by type of fracture is shown in Table S4 (the earliest mean age of subjects by type of fracture was observed in forearm and the latest mean age in hip/femur, 69.8 vs. 81.0 years).

Most subjects had a single fracture event recorded (women, 70.2% [398/567]; men, 75.5% [74/98]). Two fractures (independently of the location) were observed in 21.0% (119/567) of women and 19.4% (19/98) of men, three fractures in 6.9% (36/567) and 4.1% (4/98), respectively, and four or more fractures in 1.9% (11/567) and 1.0% (1/98) respectively. In few subjects, two femur fractures were reported.

In 175 subjects with fractures in two or more occasions, the time between the first and the second fracture was longer in women than that in men (median [95% CI]: 3.8 [2.9–4.9] vs. 2.3 [0.4–2.9] years, respectively). The survival curve in subjects aged ≥ 70 years with fractures in two or more occasions (phase B) is shown in Fig. S1.

### Pharmacological treatments

Of 661 individual prescriptions for OP medications recorded in phase B, the majority (608/661) were recorded in women. Alendronate was the most commonly prescribed OP medication for both women and men (34.9% and 28.3%), followed by denosumab (26.2% and 28.3%, respectively) (Table 4). Almost half (45.1% [298/661]) of the prescribed OP medication were discontinued, most commonly at the decision of the treating physician (31.9% [95/298]) or the subject (20.8% [62/298]), or due to tolerability issues (19.1% [57/298]).

**Table 4:** OP medication in subjects aged ≥ 70 years with fragility fracture (phase B)

	Overall treatments (*n*=661)	Women (*n*=608)	Men (*n*=53)
All OP treatments, *n* (%)			
Alendronate	227 (34.3)	212 (34.9)	15 (28.3)
Risedronate	77 (11.6)	68 (11.2)	9 (17.0)
Ibandronate	47 (7.1)	45 (7.4)	2 (3.8)
Raloxifene	16 (2.4)	16 (2.6)	0 (0.0)
Bazedoxifene	11 (1.7)	11 (1.8)	0 (0.0)
Strontium ranelate	52 (7.9)	50 (8.2)	2 (3.8)
Teriparatide	45 (6.8)	40 (6.6)	5 (9.4)
Zoledronic acid	7 (1.1)	4 (0.7)	3 (5.7)
Denosumab	174 (26.3)	159 (26.2)	15 (28.3)
Etidronate	4 (0.6)	3 (0.5)	1 (1.9)
Calcitonin	1 (0.2)	0 (0.0)	1 (1.9)
Duration of treatment (years), mean (SD)	3.6 (3.3)	3.7 (3.3)	2.5 (3.5)

*OP*, osteoporosis; *SD*, standard deviation

### Hospitalizations due to fragility fractures

Hospital admissions due to fragility fractures were reported in 29.6% (197/665) of subjects, with one hospitalization being the most frequent number (87.8% [173/197]). The most common hospitalization was due to hip/femur fracture (62.9%: 83.8% men vs. 58.8% women) (Table S5). A total of 16 patients had one hospitalization due to vertebral fracture among 230 patients with previous vertebral fracture (7.0%) (data not shown).

## Discussion

To our knowledge, this is the first study to report the prevalence of fragility fractures in individuals ≥ 70 years old seen in PC centers across different Spanish regions. We found a high prevalence of fragility fractures, with prevalence 2–3-fold higher in women than that in men and vertebral fracture the most prevalent fracture type.

Among subjects aged ≥ 70 years seen in Spanish PC centers, overall prevalence of fragility fractures was 18%. Few studies have previously reported these data, making direct comparisons with our data challenging. Using radiologic criteria, Díaz-López et al. reported prevalence of vertebral fracture between 17 and 25% in subjects ≥ 50 years from a single Spanish region [21]. In postmenopausal Spanish women aged 50–65 years, Rentero et al. reported a prevalence of 23% [22]. Prevalence of vertebral fracture (5%) in our study was much lower, most likely due to the different methods used to identify fragility fracture. In fact, unlike the study by Díaz-López et al., where the subjects were invited to participate and informed of the performance of X-rays, our study was based on the registry of vertebral fractures, probably related to some type of symptomatology. Besides, our study included other types of fractures. Furthermore, unlike previous studies, our study included both genders and it is well established that fragility fractures are more common in women than in men [23–25], mainly due to the sudden estrogen drop at menopause, lower BMD, and bone size [24] and also underdiagnosis of OP in men, who rarely undergo diagnostic tests for OP [26]. When looking at women only, the prevalence of fragility fractures (24%) observed in our study is consistent with that of previous studies from single Spanish regions [21, 27]. Using subjects’ medical records, we found vertebral fracture to be the most common fracture type, with aging and history of falls being important risk factors. These findings are to be expected, as age and history of falls are established risk factors for fragility fractures [17, 18, 28, 29]. Indeed, Díaz-López et al. reported age to be significantly associated with the presence of fracture, with fracture risk doubling with every 10-year increase in age, regardless of gender [21].

Phase B of our study found that one-third of subjects with a fragility fracture did not have a diagnosis of OP, whereas 61.1% received OP treatment at any time and more than half were not receiving any OP treatment at the time of study inclusion. These data confirm the poor management of fragility fractures in the PC setting. Several studies have reported underdiagnosis and undertreatment of OP in subjects with a previous fragility fracture in Spain [9, 13, 30, 31]. Furthermore, in a study of women aged ≥ 70 years at high risk of fragility fracture, seen in PC settings across 8 European countries, McCloskey et al. reported that most of the subjects were not receiving OP treatment and 80% did not have an OP diagnosis [13]. National and international clinical practice guidelines [18, 19] recommend treatment in subjects with fragility fractures (major fractures), regardless of BMD, or a fragility fracture if bone mass is low (osteopenia), and subjects with osteoporotic BMD without fractures. The outcomes of our study reflect the ineffective diagnosis and treatment of fragility fractures in PC. At study inclusion, approximately two-thirds of subjects had established OP; however, more than half were not receiving OP treatment, despite being elderly with high comorbidity burden and a history of previous fractures. Moreover, we found a large discrepancy between subjects who had received OP treatment and subjects who were receiving OP medication at study inclusion, suggesting there is still a high rate of discontinuation of OP medication in the PC setting. Despite differences in the age ranges evaluated, our results are consistent with previous studies. Caeiro et al. reported only 16% of subjects ≥ 65 years old hospitalized for a first hip fracture received treatment for OP at the time of the fracture [30]. Prieto-Alhambra et al. reported only 21% of subjects >50 years old with a fragility hip fracture were prescribed OP treatment at discharge [32]. Moreover, in subjects ≥ 65 years old, treatment rates fell from 29% in 2009 to 16% in 2015 [31]. In a cohort study including data from Catalonia (Spain) from 2005 to 2015, the treatment gap in subjects ≥ 50 years old was 80–88% [14]. Based on the database for pharmaco-epidemiological research in PC 2011, León Vásquez et al. reported 39% of subjects ≥ 60 years old received OP treatment after the fracture [33]. Collectively, these data indicate that management of OP among high-risk subjects seen in PC can be improved.

Our study has some limitations. Due to the observational and retrospective design, there were missing data. Moreover, 2.2–10-fold differences in the prevalence of vertebral fracture have been reported, depending on the radiological criteria [21, 34–36]. The ICD codes used in our study could add variability in the observed prevalence. Also, fractures can be asymptomatic [10] and the reported prevalence could be underestimated. Conversely, some fractures may have been incorrectly identified as fragility fractures; however, a recent study of more than 300 fractures coded in subjects aged >50 years old reported that >90% of hip fractures, >87% of vertebral fractures, and >80% of major fractures were fragility/osteoporotic (i.e., not related to high-impact trauma) [29]. Finally, among all PC centers invited to participate in the study, PC centers that accepted to participate were those with interest and/or experience in OP. This is a study bias of the medical center selection that could overestimate the OP diagnosis and biased the characteristic of the subjects including treatment rates compared to PC centers without OP experience. This fact shows that the participating PC centers have the potential to do better in terms of diagnosis and treatment. Moreover, our study included centers from different Spanish regions (specifically, 73.7% of the Spanish regions), providing a representative sample of most of the Spanish regions of subjects ≥ 70 years old (at the time of data collection).

## Conclusion

We observed a high prevalence of fragility fractures among the elderly (≥ 70 years) subjects seen in Spanish PC, and a large diagnosis and treatment gap among subjects with a fragility fracture. Compared to women, these gaps were larger in men. Our data highlight the urgent need to improve the management of fragility fractures in PC. As PC centers participating in this study had high OP experience that have the potential to do better in terms of diagnosis and treatment, caution in the generalization of these data should be taken.

## Supplementary Information

Below is the link to the electronic supplementary material.

Supplementary file1 (DOCX 30 KB)

Supplementary file2 (JPG 46 KB)

## Data Availability

All data relevant to the study are included in the article or uploaded as supplemental information.
