# Supplementary material for: Prevalence and treatment of fragility fractures in Spanish primary care: PREFRAOS study
Source: Arch Osteoporos. 2022 Jul 15;17(1):93. doi: 10.1007/s11657-022-01124-7 (PMC9283348; doi:10.1007/s11657-022-01124-7)
Supplement: Supplementary file 1 — Supplementary file1 (DOCX 30 KB) [file 11657_2022_1124_MOESM1_ESM.docx]

**Sup Table 1**  Number of OP medications (Phase B)

| **Percentage of subjects with OP pharmacological treatments (%)** | **Women**  **(n=576)** | **Men**  **(n=98)** | **Overall**  **(n=665)** |
| --- | --- | --- | --- |
| 1 | 61.8 | 64.9 | 62.1 |
| 2 | 21.4 | 27.0 | 21.9 |
| 3 | 10.3 | 8.1 | 10.1 |
| ≥4 | 6.5 | 0.0 | 5.9 |

**Sup Table 2** Current OP medication by OP diagnosis

| **Percentage of subjects (%)** | **No OP diagnosis**  **(n=228)** | **OP diagnosis**  **(n=437)** | **Overall**  **(n=665)** |
| --- | --- | --- | --- |
| Yes | 13.2 | 58.8 | 43.2 |
| No | 86.8 | 41.2 | 56.8 |

**Sup Table 3** Most common types of fragility fractures (Phase B)

| **Percentage of subjects (%)** | **Fractures in women (n=800)** | **Fractures in men**  **(n=128)** | **Overall fractures**  **(n=928)** |
| --- | --- | --- | --- |
| Vertebral | 33.0 | 32.0 | 32.9 |
| Humerus | 12.8 | 9.4 | 12.3 |
| Radius/Ulna | 10.5 | 4.7 | 9.7 |
| Wrist | 8.9 | 3.9 | 8.2 |
| Pelvis | 4.5 | 1.6 | 4.1 |
| Rib | 2.8 | 6.3 | 3.2 |
| Ankle/Foot | 3.4 | 1.6 | 3.1 |
| Tibia/Fibula | 2.4 | 0.8 | 2.2 |
| Patella | 1.5 | 0.0 | 1.1 |
| Clavicle | 0.8 | 1.6 | 0.9 |
| Other | 1.5 | 0.0 | 1.3 |

**Sup Table 4** Mean age of subjects when fragility fractures occurred by type of fracture (Phase B)

| **Mean age of subjects at fracture (years)** | **Fractures in women (n=800)** | **Fractures in men**  **(n=128)** | **Overall fractures**  **(n=928)** |
| --- | --- | --- | --- |
| Forearm | 69.7 | 70.9 | 69.8 |
| Hip/Femur | 80.4 | 82.7 | 81 |
| Clavicle | 79.8 | 56.2 | 73.9 |
| Rib | 78.3 | 75.5 | 77.6 |
| Humerus | 76.4 | 81.2 | 76.9 |
| Wrist | 72.8 | 78.2 | 73.2 |
| Pelvis | 79.8 | 77.2 | 79.6 |
| Radius/Ulna | 73.7 | 80.5 | 74.2 |
| Patella | 75.4 | - | 75.4 |
| Tibia/Fibula | 70.8 | 69.9 | 70.7 |
| Ankle/Foot | 71.6 | 78.3 | 72.1 |
| Vertebral | 76.0 | 76.6 | 76.1 |

**Sup Table 5**  Percentage of subjects hospitalized due to fragility fractures by type of fracture (Phase B)

| **Percentage of subjects hospitalized (%)** | **Women (n=187)** | **Men (n=37)** | **Overall (n=224)** |
| --- | --- | --- | --- |
| Hip/Femur | 58.8 | 83.8 | 62.9 |
| Humerus | 13.4 | 0.0 | 11.2 |
| Pelvis | 4.8 | 0.0 | 4.0 |
| Tibia/Fibula | 5.3 | 0.0 | 4.5 |
| Vertebral | 5.9 | 13.5 | 7.1 |
| Wrist/Forearm | 7.5 | 2.7 | 6.7 |
| Others | 4.3 | 0.0 | 3.6 |
