# Supplementary figures and images for: Prevalence and treatment of fragility fractures in Spanish primary care: PREFRAOS study
Source: Arch Osteoporos. 2022 Jul 15;17(1):93. doi: 10.1007/s11657-022-01124-7 (PMC9283348; doi:10.1007/s11657-022-01124-7)

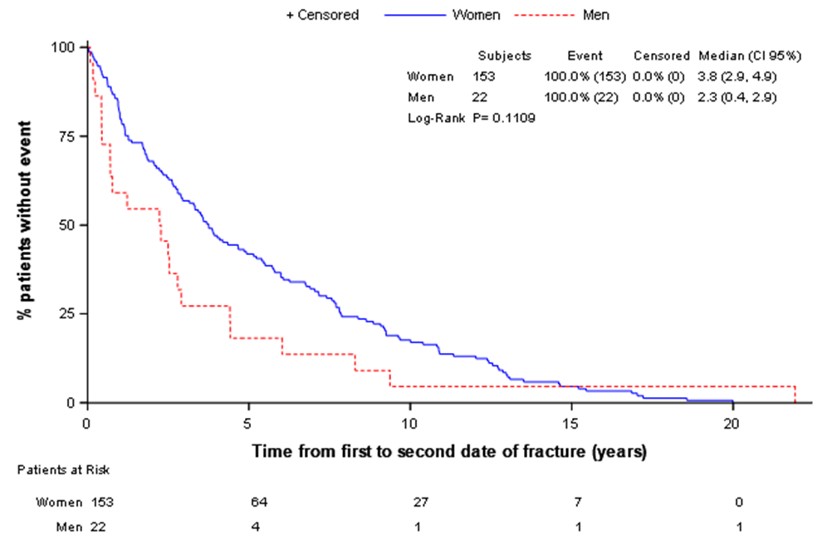

Supplement: Supplementary file 2 — Supplementary file2 (JPG 46 KB) [file 11657_2022_1124_MOESM2_ESM.jpg]
